# Papillary thyroid microcarcinoma: characteristics at presentation, and evaluation of clinical and histological features associated with a worse prognosis in a Latin American cohort

**DOI:** 10.20945/2359-3997000000013

**Published:** 2018-01-01

**Authors:** José M. Domínguez, Flavia Nilo, María T. Martínez, José M. Massardo, Sueli Muñoz, Tania Contreras, Rocío Carmona, Joaquín Jerez, Hernán González, Nicolás Droppelmann, Augusto León

**Affiliations:** 1 Pontificia Universidad Católica de Chile Pontificia Universidad Católica de Chile Faculty of Medicine Department of Endocrinology Santiago Chile Department of Endocrinology, Faculty of Medicine, Pontificia Universidad Católica de Chile, Santiago, Región Metropolitana, Chile; 2 Pontificia Universidad Católica de Chile Pontificia Universidad Católica de Chile Faculty of Medicine Department of Surgery Santiago Chile Department of Surgery, Faculty of Medicine, Pontificia Universidad Católica de Chile, Santiago, Región Metropolitana, Chile

**Keywords:** Thyroid cancer, head and neck, endocrine

## Abstract

**Objective:**

We aimed to describe the presentation of papillary microcarcinoma (PTMC) and identify the clinical and histological features associated with persistence/recurrence in a Latin American cohort.

**Subjects and methods:**

Retrospective study of PTMC patients who underwent total thyroidectomy, with or without radioactive iodine (RAI), and who were followed for at least 2 years. Risk of recurrence was estimated with ATA 2009 and 2015 classifications, and risk of mortality with 7^th^ and 8^th^ AJCC/TNM systems. Clinical data obtained during follow-up were used to detect structural and biochemical persistence/recurrence.

**Results:**

We included 209 patients, predominantly female (90%), 44.5 ± 12.6 years old, 183 (88%) received RAI (90.4 ± 44.2 mCi), followed-up for a median of 4.4 years (range 2.0–7.8). The 7^th^ and 8^th^ AJCC/TNM system classified 89% and 95.2% of the patients as stage I, respectively. ATA 2009 and ATA 2015 classified 70.8% and 78.5% of the patients as low risk, respectively. Fifteen (7%) patients had persistence/recurrence during follow-up. In multivariate analysis, only lymph node metastasis was associated with persistence/recurrence (coefficient beta 4.0, p = 0.016; 95% CI 1.3-12.9). There were no PTMC related deaths.

**Conclusions:**

Our series found no mortality and low rate of persistence/recurrence associated with PTMC. Lymph node metastasis was the only feature associated with recurrence in multivariate analysis. The updated ATA 2015 and 8^th^ AJCC/TNM systems classified more PTMCs than previous classifications as low risk of recurrence and mortality, respectively.

## INTRODUCTION

Papillary thyroid microcarcinoma (PTMC) is defined as a papillary thyroid cancer (PTC) whith a diameter 1 cm or less, and its incidence has increased during the last decades (1,2). PTMC comprises nearly 50% of PTC diagnosed worldwide and is associated with a favorable prognosis, which varies depending on the extension of the disease. Intrathyroidal incidental PTMC has the best prognosis, with recurrence and mortality rates as low as 0.5% and 0%, respectively (3-6). On the other hand, the 1-2.8% of patients with PTMC who have distant metastases at diagnosis have a worse prognosis, with high rates of persistence/recurrence (7). Previous studies have associated multifocality, bilateral disease, lymph node (LN) metastasis, tumor diameter of > 0.5 cm, a sum of multifocal tumor diameters greater than 2 cm, and minimal extra thyroidal extension (ETE) with a higher risk of recurrence (8-10).

Due to its good prognosis, some experts have proposed renaming PTMC as “micropapillary tumor” thus avoiding the word “cancer” and its implications regarding overtreatment and the deterioration of patient's quality of life (11,12). Additionally, the favorable prognosis of PTMC has suggested the need for a less intense treatment with regard to surgery extension, radioactive iodine (RAI) ablation and TSH suppression, without increasing recurrence and mortality (13). Furthermore, active surveillance of selected PTMC patients has shown promising results, with a low risk of enlargement and LN metastases after 10 years of follow-up (14).

These less intense strategies for PTMC treatment are consistent with the updated versions of the 2015 American Thyroid Association (ATA) guidelines and the 8^th^ AJCC/TNM system, which suggest changes in the stratification of the risks of recurrence and mortality, respectively (8,13,15). The most important changes include the following: i) the impact of LN metastases is now said to depend on both their number and size but not on their sole presence; ii) the role of minimal ETE has been reduced; and; iii) the age that determines a higher risk of mortality has been increased from 45 to 55 years (15,16).

Although PTMC represents nearly 50% of PTC patients, there is scarce information on this subject in relation to the Latin American population (5,17-19). Our aim was to describe the characteristics of PTMC at diagnosis and to identify the clinical and histological features associated with persistence/recurrence in a Latin American cohort. We hypothesized that our patients would have low rates of recurrence and mortality. We also expected that the updated ATA 2015 classification and the 8^th^ AJCC/TNM system would induce significant changes in both recurrence and mortality risk stratification, respectively.

## SUBJECTS AND METHODS

After receiving the approval of the Ethics Committee, we retrospectively reviewed the electronic medical records of 673 consecutive patients with PTC who were 18 years old or older, who had undergone total thyroidectomy between 2009 and 2013, and who had been followed-up for at least 2 years. We included every patient with PTMC. LN dissection was performed in patients whose preoperative ultrasound, fine needle aspiration biopsy, or intra-operative findings, suggested LN metastases. We used fixed RAI activities based on the extent of the initial disease, and the dose administered was decided by the attending physician, following ATA 2009 and institutional guidelines (8,20). A post-therapy whole body scan (WBS) was performed after RAI. Considering the results of preoperative ultrasound, intra-operative findings, pathology and WBS result, the patients were risk-stratified using the 7^th^ and 8^th^ edition of the AJCC/UICC staging system and the ATA 2009 and ATA 2015 risk of recurrence stratification systems (8,13). Of the 673 potentially eligible patients, a total of 209 patients satisfied the inclusion criteria.

After initial therapy, all patients received levothyroxine to keep TSH between 0.5 and 1.0 uUI/mL, and had at least two neck ultrasounds and two serum thyroglobulin (Tg) and anti-Tg antibodies (TgAb) determinations, either suppressed or stimulated by levothyroxine withdrawal.

Patients were routinely followed every 6 months during the first year, and at 6–12 month intervals thereafter at the discretion of the attending physician, based on the risk of the individual patient and the clinical course of the disease.

Structural neck persistent/recurrent PTC was defined as suspicious LN in neck ultrasound, accompanied by histocytological or Tg in an aspirate study that proved the presence of PTC. Extra-cervical structural persistent/recurrent PTC was defined as the presence of suspicious images on cross-sectional studies (computed tomography scan, or magnetic resonance imaging) or functional imaging (diagnostic WBS or ^18^FDG PET-scan), requested according to the attending physician criteria. Biochemical persistent/recurrent PTC was defined as the presence of suppressed 1^st^ generation Tg ≥ 0.9 ng/mL or stimulated Tg ≥ 2.0 ng/mL, without structural disease. At final followup, the absence of abnormal images in radiological studies accompanied by stimulated Tg < 2.0 ng/mL or suppressed 1^st^ generation Tg < 0.9 ng/mL, were considered no clinical evidence of disease (NED).

Tg was measured using a chemiluminescent assay (Immulite 2000, Siemens, Los Angeles, CA, USA) with a functional sensitivity of 0.9 ng/mL, normalized to CRM 457. TgAb were measured using a chemiluminescent assay (Architect i1000, Laboratories, Abbott Park, IL, USA) (reference value of up to 4.11 IU/mL, analytical sensitivity 1.0 IU/mL). Since May 12^th^, 2014, second generation Tg was measured using a chemiluminescent assay (Elecsys II, Roche Diagnostics, Rotkreuz, Switzerland) with a functional sensitivity of 0.1 ng/mL, normalized to CRM 457.

Categorical variables are expressed as number and frequencies; continuous variables are expressed as mean ± SD, or median and ranges, as appropriate. Proportions were compared using a Fisher's exact chi2 test. Continuous variables were compared using parametric or non-parametric tests, as appropriate. Analysis was performed using SPSS software (version 15.0.0: SPSS, Inc., Chicago, IL). P-values < 0.05 were considered to be statistically significant.

## RESULTS

Patients were predominantly female (90%) and 44.5 ± 12.6 years old: A total of 183 (88%) received RAI (90.4 ± 44.2 mCi) and were followed-up for a median of 4.4 years (range 2.0–7.8 years). Most patients (78%) had classical variant PTC, 61.2% had unifocal, 76.1% had unilateral disease, 17.9% had minimal ETE (microscopic or involving strap muscles), 16.7% had LN metastasis and none had distant metastases at diagnosis (Table 1).

**Table 1:** Characteristics of the patients at baseline

		Total Cohort (N = 209)	Persistence/Recurrence		P-value
			No (n = 194)	Yes (n = 15)	
Age at diagnosis (years)		44.5 ± 12.6	44.7 ± 12.8	40.8 ± 9.4	0.08
Female gender		188 (90%)	173 (89.1%)	15 (100%)	0,18
Histological subtype of PTC					0.6
	Classical variant	163 (78%)	150 (77.3%)	13 (87.0%)	
	Follicular variant	36 (17.2%)	34 (17.7%)	2 (13.0%)	
	Other variants	10 (4.8%)	10 (5.130%)	0 (0%)	
Tumor diameter (cm)		0.62 ± 0.25	0,62 ± 0.26	0.65 ± 0.25	0.75
Sum of diameters (cm)		0.83 ± 0.54	0.83 ± 0.55	0.9 ± 0.48	0.9
Sum of all foci diameters > 2 cm		10 (4.8%)	10 (5.1%)	0 (0%)	0.36
Unifocal disease		128 (61.2%)	121 (62.4%)	7 (47.0%)	0.3.
Three or more foci		35 (16.7%)	30 (15.5%)	5 (33.3%)	0.07
Bilateral disease		50 (23.9%)	46 (23.7%)	4 (26.7%)	0.80
Minimal extra thyroidal extension		37 (17.7%)	31 (16.0%)	6 (40.0%)	0.019
Lymphocytic thyroiditis		88 (42.1%)	83 (42.8%)	5 (33.3%)	0.37
T					0.33
	1a	172 (82.3%)	154 (82.8%)	8 (61.5%)	
	3	37 (17.7%)	32 (17.2%)	5 (38.5%)	
N*					0.001
	0	174 (83.3%)	166 (86.0%)	8 (53.3%)	
	1	35 (16.7%)	28 (14.0%)	7 (46.7%)	
RAI ablation		183 (88.0%)	168 (86.6%)	15 (100%)	0.40
RAI dose (mCi)		90.4 ± 44.2	88.2 ± 44.2	116.7 ± 36.2	0.69
Length of follow-up (years)		4.4 (2.0-7.8)	4.4 (2.0 – 7.8)	4.2 (2.1 – 6.3)	0.37
AJCC 7^th^					
	I	186 (89%)	173 (89.2%)	13 (86.6%)	0.06
	III	21 (10%)	20 (10.3%)	1 (6.7%)	
	IVA	2 (1.0%)	1 (0.5%)	1 (6.7%)	
AJCC 8^th^					0.34
	I	199 (95.2%)	184 (95.0%)	15 (100%)	
	II	10 (4.8%)	10 (5.0%)	0 (0%)	
ATA 2009					0.006
	Low	148 (70.8%)	142 (73.2%)	6 (40%)	
	Intermediate	61 (29.2%)	52 (26.8%)	9 (60%)	
ATA 2015					0.002
	Low	164 (78.5%)	157 (81%)	7 (46.7%)	
	Intermediate	45 (21.5%)	37 (19%)	8 (53.3%)	

* N0 includes patients subjected to lymph node dissection whose biopsy found no tumor, and in whom physical exam, preoperative ultrasound and intraoperative evaluation found no evidence of lymph node metastases.

According to the 7^th^ AJCC/TNM system, 89%, 10% and 1% of the patients were classified as stage I, stage III and stage IVA disease, respectively. Using the updated 8^th^ AJCC/TNM system, 95.2% and 4.8% of the patients were classified as stage I and stage II disease, respectively, with no patients classified as stage III or stage IVA. Regarding risk of recurrence, ATA 2009 classified 70.8% and 29.2% of the patients as low and intermediate risk, respectively, while ATA 2015 categorized 78.5% and 21.5% of the patients as low and intermediate risk, respectively (Table 1).

Of the whole cohort, 194 (93%) patients had no persistence/recurrence, while 3 (1.5%) and 12 (5.5%) had biochemical and structural persistence/recurrence, respectively. There were no PTMC related deaths and the characteristics of patients who recurred are shown in Tables 1 and 2.

**Table 2:** Characteristics of 15 patients with persistent or recurrent disease

Patient number	ATA 2015	Type of persistence/recurrence	Time between initial treatment and to persistence/recurrence (years)	Preoperative neck ultrasound done	Serum Tg (ng/mL)		Follow-up neck US	FNA	Therapy	2^nd^ Gen Tg (ng/mL)	Length of follow-up (years)	Status at end of follow-up
					Suppressed	Stimulated						
1837	Low	Structural	0.8	No	< 0.9	NA	(+)	(+)	Sx	< 0.1	6.8	NED
1897	Low	Structural	2.2	No	3.6	NA	(+)	(+)	Sx + RAI	< 0.1	6.7	NED
2015	Intermediate	Structural	1.4	No	10.6	NA	(+)	NA	RAI	0.7	3.9	Biochemical disease
1952	Low	Structural	0.9	No	3.4	NA	(+)	(+)	Sx + RAI	< 0.1	6.1	NED
1997	Low	Structural	5.81	No	0.1	3.2	(-)	Chest CT and Dx scan compatible with 1.7 cm pulmonary metastasis	Sx + RAI	0.1	6.5	NED
1511	Low	Structural	1.2	No	5.5	NA	(+)	(+)	Sx + RAI	< 0.1	7.5	NED
1732	Intermediate	Structural	1.0	No	1.2	33	(+)	(+)	Sx + RAI	6.5	0.2	NED
2172	Intermediate	Structural	0.6	No	5.0	NA	(+)	(+)	Sx	4.8	<0.1	NED
2174	Intermediate	Structural	0.7	No	3.8	21	(+)	(+)	Sx	5.0	0.2	NED
2315	Low	Structural	1.96	Negative	< 0.9	NA	(+)	(+)	AS	0.2	4.5	Neck US non-specific nodule (granuloma) + negative chest CT
2232	Intermediate	Structural	4.0	No	< 0.9	NA	1 cm suspicious lateral cervical lymph node	Not done	AS	< 0.1	4.9	Stable neck US
2296	Intermediate	Structural	1.8	Cervical lymph nodes (+)	1.0	1.8	(+)	(+)	Sx + RAI	< 0.1	4.3	NED
1506	Low	Biochemical	0.9	Negative	2.4	27.1	(-)	(-)	AS	1.0	6.9	Biochemical disease (negative neck US and ^18^FDG PET- CT)
1520	Intermediate	Biochemical	2.4	Cervical lymph nodes (+)	< 0.9	2.8	(-)	NA	AS	< 0.1	7.5	NED
2195	Intermediate	Biochemical	1.1	Negative	< 0.9	2.7	(-)	NA	AS	0.2	4.8	NED Neck US + Chest CT negative

Tg: thyroglobulin; NA: not applicable; Sx: surgery; RAI: radioactive iodine; NED: not evidence of disease; CT: computed tomography; Dx scan: ^131^I diagnostic scan; AS: active surveillance; US: ultrasound.

In univariate analysis, minimal ETE (OR 3.5 (95% CI 1.2-10.6)) and cervical LN metastasis (OR 5.2 (95% CI 1.7-15.4)) were associated with persistence/recurrence (Table 1). In multivariate analysis, only LN metastasis kept its significance (beta coefficient 4.0 (p = 0.016; 95% CI 1.3-12.9)).

The characteristics of the 15 patients who had persistence/recurrence are detailed in Table 2. Preoperative neck ultrasound was performed in only 5 (33%) of them. The median time between initial treatment and persistence/recurrence was 1.2 years (range 0.8-5.8) and 9 cases (60%) were diagnosed earlier than 1.5 years after initial treatment. Among the 12 patients with structural persistence/recurrence, 11 had cervical LN metastases: 5 underwent surgery and RAI, 3 underwent surgery alone, 1 received RAI alone and 2 underwent active surveillance: at the time of analysis, none of the patients showed progression of structural disease, while 7 accomplished a 2^nd^ generation Tg ≤ 0.2 ng/dL, consistent with an excellent response to treatment.

The additional patient with structural disease was a 43-year old woman who had been treated initially with total thyroidectomy and RAI ablation (50 mCi) for a low risk PTMC. She achieved an excellent response (negative neck ultrasound and serum Tg of 0.1 ng/dL), but a 1.7 cm pulmonary metastasis was incidentally diagnosed on a chest X-ray 5.8 years after initial treatment. The lung metastasis was resected, an additional 100 mCi dose of RAI was given and the patient currently has NED.

Of the 3 patients with biochemical persistence/recurrence, all three underwent active surveillance. At the end of the follow-up, none had evidence of structural disease and 2 had achieved Tg ≤ 0.2 ng/dL, demonstrating an excellent response.

Of 194 patients with no persistence/recurrence, 2^nd^ generation Tg was obtained in 146: 143 (98%) had Tg ≤ 0.2 ng/mL, while 3 patients had values between 0.3 and 0.9 ng/mL. None of them developed structural disease.

We found no significant differences for gender, histological subtypes, rate of multifocal disease, ETE, TNM/AJCC system, or risk of recurrence classifications among patients who had received and those who had not received RAI.

## DISCUSSION

In this study, in which we evaluated a cohort of 209 Latin American patients, PTMC was associated with good prognosis: there were no PTMC related deaths, 7% of patients had persistence/recurrence, and 204 (98%) had NED at the end of the follow-up. These results are consistent with previously published series, which report mortality and recurrence rates of between 0-3.0% and 0.5-7.9%, respectively (9).

Previous reports have shown that non-incidental PTMC has higher risk of recurrence than incidental PTMC (6). The patients included in our series have a similar profile as those with non-incidental PTMC according to the previous series: predominantly female, 44.5 ± 12.6 years old, tumor diameter 0.62 ± 0.25 cm, multifocality (38.8%), bilaterality (23.9%), minimal ETE (17.7%), and LN metastasis (16.7%) (6). This similarity to non-incidental PTMC may explain why the recurrence rate was close to the highest reported.

Published data reported rates of LN metastases between 10-50%, with the highest rates found when prophylactic central LN dissection is performed (21). In our series, only 16.7% of PTMC had LN metastases, which is probably due to our policy of performing therapeutic neck dissection exclusively in patients with clinical evidence of N1 disease. This might also explain the impact of LN metastases in persistence/recurrence after multivariate analysis. It is currently known that the impact of LN metastases on recurrence depends on the number and size of the metastases, and on the presence of extra-nodal extension (8). Although it was controversial in the past, retrospective studies have not found lower rates of recurrence in PTC patients subjected to prophylactic central neck dissection, but may be associated with higher risks of complications (22,23).

In our series, minimal ETE had no impact on recurrence/persistence. Although it may be controversial and ATA 2015 guidelines still consider its presence to be criterion with which to classify patients as being at intermediate risk of recurrence, our findings are consistent with other studies that found no association with recurrence (10,24). Furthermore, the updated 8^th^ TNM/AJCC system discouraged its role as a predictor of high risk of mortality in PTC, and recent data has shown poor agreement among expert pathologists in terms of defining its presence (15,25).

As patients were treated following older guidelines, 183 (93%) patients received RAI: they had similar characteristics at presentation to those who were not ablated, and RAI had no impact on recurrence/persistence, which is consistent with ATA 2015 recommendations and other series (13,26). We also recognize that currently a significant number of these patients would not undergo total thyroidectomy, as PTMC prognosis is similar in patients treated with partial thyroidectomy (13,27).

In our series, most patients with persistence/recurrence disease, 11 of 15, had structural regional disease. When analyzing their characteristics, only one third of them had preoperative neck ultrasound, and in two thirds persistence/recurrence was diagnosed within 1.5 years of initial treatment. These two facts suggest that the incomplete preoperative stratification is an explanation for the increased rate of structural persistent disease found early on in the follow-up. They also emphasize that the need for a comprehensive preoperative ultrasound staging in patients with PTMC is as important as it is in non-PTMC patients (28). From 2012 onwards, preoperative ultrasound staging was established as part of our routine protocol for every patient with diagnosis of thyroid cancer.

Of the 11 patients with cervical persistent/recurrence, 9 were actively treated with surgery, RAI, or a combination of both. The remaining 2 patients underwent active surveillance: one had NED and the other had stable findings at the end of the follow-up. This finding supports a less aggressive management of low volume cervical LN disease in properly selected patients, which has been previously associated with 5-10% risks of local progression, without or without very low risk of distant metastases (29,30).

Only one patient developed a distant metastasis during follow-up, which was incidentally discovered in a chest X-ray 5.81 years after initial treatment with total thyroidectomy and RAI. The patient had had a long period of excellent response, making recurrence very unlikely. After a lung metastasis resection and an additional RAI dose, the patient currently has NED.

An additional interesting finding is the impact of the updated risk of mortality and recurrence classifications. Regarding recurrence, in our series the rate of low risk patients rose from 70.8% to 78.5% according to ATA 2009 and 2015 classifications, respectively, which is consistent with the low rates of recurrence found in PTMC (8,13). Based on the recently published 8^th^ AJCC/TNM system, the rate of stage I disease increased to 95%, and none of the patients were classified as stage III (8,13,15). These two changes are consistent with the very low risk of death related to PTMC in our and others series (6,16,31). These improvements in the stratification of PTMC patients should allow physicians to better adjust the intensity of therapy and avoid overtreatment in most patients with PTMC, which has been associated with side effects and deterioration of quality of life (9). Even more, in properly selected patients, active surveillance has shown to be feasible, with low risk of progression of disease and lower rates of side effects related to therapy (14,32).

A limitation of this study is the short time of followup, a median of 4.4 years (range 2.0-7.8). However, although PTC may recur a long time after initial treatment, nearly 50% and up to 80% of the recurrences occur during the first 3 and 5 years after initial treatment, respectively (33). In addition, it is important to note that among the 194 patients who did not have persistence/recurrence, 2^nd^ generation Tg was available in 146: 143 had Tg ≤ 0.2 ng/mL, which is consistent with an excellent response to treatment and a very low risk of recurrence (13,34,35). The remaining 3 patients had negative imaging studies and stable serum Tg of between 0.3 and 0.9ng/mL, which is consistent with an indeterminate response, a category that still has good prognosis, with less than 1% risk of death and nearly 15% risk of structural progression (13).

In conclusion, we found a low rate of recurrence and no mortality associated with PTMC in Latin American patients. LN metastasis was the only feature associated with higher risk of recurrence, which emphasizes the importance of a comprehensive preoperative neck ultrasound evaluation. Updated ATA 2015 and 8^th^ AJCC/TNM systems had a significant impact on patient stratification by increasing the rate of patients classified as at low risk of recurrence and mortality, respectively.

## References

[1] 1. Hedinger C, Williams ED, Sobin LH. The WHO histological classification of thyroid tumors: a commentary on the second edition. Cancer. 1989;63:908-11. 10.1002/1097-0142(19890301)63:5<908::aid-cncr2820630520>3.0.co;2-i 2914297

[2] 2. Davies L, Welch HG. Current thyroid cancer trends in the United States. JAMA Otolaryngol Head Neck Surg. 2014;140:317-22. 10.1001/jamaoto.2014.1 24557566

[3] 3. Lee YS, Lim H, Chang HS, Park CS. Papillary thyroid microcarcinomas are different from latent papillary thyroid carcinomas at autopsy. J Korean Med Sci. 2014;29:676-9. 10.3346/jkms.2014.29.5.676 PMC4024958 24851024

[4] 4. Hughes DT, Haymart MR, Miller BS, Gauger PG, Doherty GM. The most commonly occurring papillary thyroid cancer in the United States is now a microcarcinoma in a patient older than 45 years. Thyroid. 2011;21:231-6. 10.1089/thy.2010.0137 21268762

[5] 5. Mosso L, Campusano C, González H, Domínguez JM, Salman P, Suazo V, et al. [From macro to micro thyroid carcinoma: records of a clinical hospital from 1991 to 2010]. Rev Med Chil. 2013;141:442-8. 10.4067/S0034-98872013000400004 23900364

[6] 6. Mehanna H, Al-Maqbili T, Carter B, Martin E, Campain N, Watkinson J, et al. Differences in the recurrence and mortality outcomes rates of incidental and nonincidental papillary thyroid microcarcinoma: a systematic review and meta-analysis of 21 329 person-years of follow-up. J Clin Endocrinol Metab. 2014;99:2834-43. 10.1210/jc.2013-2118 24828487

[7] 7. Bernet V. Approach to the patient with incidental papillary microcarcinoma. J Clin Endocrinol Metab. 2010;95:3586-92. 10.1210/jc.2010-0698 20685885

[8] 8. American Thyroid Association (ATA) Guidelines Taskforce on Thyroid Nodules and Differentiated Thyroid Cancer, Cooper DS, Doherty GM, Haugen BR, Kloos RT, Lee SL, Mandel SJ, et al. Revised American Thyroid Association management guidelines for patients with thyroid nodules and differentiated thyroid cancer. Thyroid. 2009;19:1167-214. 10.1089/thy.2009.0110 19860577

[9] 9. Leboulleux S, Tuttle RM, Pacini F, Schlumberger M. Papillary thyroid microcarcinoma: time to shift from surgery to active surveillance? Lancet Diabetes Endocrinol. 2016;4:933-42. 10.1016/S2213-8587(16)30180-2 27550849

[10] 10. Kluijfhout WP, Pasternak JD, Kwon JS, Lim J, Shen WT, Gosnell JE, et al. Microscopic Positive Tumor Margin Does Not Increase the Risk of Recurrence in Patients with T1-T2 Well-Differentiated Thyroid Cancer. Ann Surg Oncol. 2016;23:1446-51. 10.1245/s10434-015-4998-x 26628431

[11] 11. Rosai J, LiVolsi VA, Sobrinho-Simoes M, Williams ED. Renaming papillary microcarcinoma of the thyroid gland: the Porto proposal. Int J Surg Pathol. 2003;11:249-51. 10.1177/106689690301100401 14615819

[12] 12. Rosario PW. Papillary microtumor or papillary microcarcinoma of the thyroid? A prospective analysis of the Porto Proposal. Int J Surg Pathol. 2013;21:639-40. 10.1177/1066896913502232 23999116

[13] 13. Haugen BR, Alexander EK, Bible KC, Doherty GM, Mandel SJ, Nikiforov YE, et al. 2015 American Thyroid Association Management Guidelines for Adult Patients with Thyroid Nodules and Differentiated Thyroid Cancer: The American Thyroid Association Guidelines Task Force on Thyroid Nodules and Differentiated Thyroid Cancer. Thyroid. 2016;26:1-133. 10.1089/thy.2015.0020 PMC4739132 26462967

[14] 14. Ito Y, Miyauchi A, Kihara M, Higashiyama T, Kobayashi K, Miya A. Patient age is significantly related to the progression of papillary microcarcinoma of the thyroid under observation. Thyroid. 2014;24:27-34. 10.1089/thy.2013.0367 PMC3887422 24001104

[15] 15. Tarasova VD, Tuttle RM. Current Management of Low Risk Differentiated Thyroid Cancer and Papillary Microcarcinoma. Clin Oncol (R Coll Radiol). 2017;29:290-7. 10.1016/j.clon.2016.12.009 28087101

[16] 16. Nixon IJ, Wang LY, Migliacci JC, Eskander A, Campbell MJ, Aniss A, et al. An International Multi-Institutional Validation of Age 55 Years as a Cutoff for Risk Stratification in the AJCC/UICC Staging System for Well-Differentiated Thyroid Cancer. Thyroid 2016;26:373-80. 10.1089/thy.2015.0315 PMC4790212 26914539

[17] 17. Monteros Alvi M, Gonorazky S, Virgili E, Soler G, Fernández M, van Cauwlaert L. [Non-incidental papillary microcarcinomas of the thyroid]. Medicina (B Aires). 2008;68:139-43. 18499963

[18] 18. Cordioli MI, Canalli MH, Coral MH. Increase incidence of thyroid cancer in Florianopolis, Brazil: comparative study of diagnosed cases in 2000 and 2005. Arq Bras Endocrinol Metabol. 2009;53:453-60. 10.1590/s0004-27302009000400011 19649384

[19] 19. Friguglietti CU, Dutenhefner SE, Brandao LG, Kulcsar MA. Classification of papillary thyroid microcarcinoma according to size and fine-needle aspiration cytology: Behavior and therapeutic implications. Head Neck. 2011;33:696-701. 10.1002/hed.21517 20737499

[20] 20. Pitoia F, Ward L, Wohllk N, Friguglietti C, Tomimori E, Gauna A, et al. Recommendations of the Latin American Thyroid Society on diagnosis and management of differentiated thyroid cancer. Arq Bras Endocrinol Metabol. 2009;53:884-7. 10.1590/s0004-27302009000700014 19942992

[21] 21. Qu N, Zhang L, Ji QH, Chen JY, Zhu YX, Cao YM, et al. Risk Factors for Central Compartment Lymph Node Metastasis in Papillary Thyroid Microcarcinoma: A Meta-Analysis. World J Surg. 2015;39:2459-70. 10.1007/s00268-015-3108-3 26099728

[22] 22. Moreno MA, Edeiken-Monroe BS, Siegel ER, Sherman SI, Clayman GL. In papillary thyroid cancer, preoperative central neck ultrasound detects only macroscopic surgical disease, but negative findings predict excellent long-term regional control and survival. Thyroid. 2012;22:347-55. 10.1089/thy.2011.0121 PMC3968956 22280230

[23] 23. Wada N, Duh QY, Sugino K, Iwasaki H, Kameyama K, Mimura T, et al. Lymph node metastasis from 259 papillary thyroid microcarcinomas: frequency, pattern of occurrence and recurrence, and optimal strategy for neck dissection. Ann Surg. 2003;237:399-407. 10.1097/01.SLA.0000055273.58908.19 PMC1514312 12616125

[24] 24. Hay ID, Johnson TR, Thompson GB, Sebo TJ, Reinalda MS. Minimal extrathyroid extension in papillary thyroid carcinoma does not result in increased rates of either cause-specific mortality or postoperative tumor recurrence. Surgery 2016;159:11-9. 10.1016/j.surg.2015.05.046 26514317

[25] 25. Su HK, Wenig BM, Haser GC, Rowe ME, Asa SL, Baloch Z, et al. Inter-Observer Variation in the Pathologic Identification of Minimal Extrathyroidal Extension in Papillary Thyroid Carcinoma. Thyroid. 2016;26:512-7. 10.1089/thy.2015.0508 PMC5583558 26953223

[26] 26. Kwon H, Jeon MJ, Kim WG, Park S, Kim M, Kim TY, et al. Lack of Efficacy of Radioiodine Remnant Ablation for Papillary Thyroid Microcarcinoma: Verification Using Inverse Probability of Treatment Weighting. Ann Surg Oncol. 2017;24:2596-602. 10.1245/s10434-017-5910-7 28600731

[27] 27. Donatini G, Castagnet M, Desurmont T, Rudolph N, Othman D, Kraimps JL. Partial Thyroidectomy for Papillary Thyroid Microcarcinoma: Is Completion Total Thyroidectomy Indicated? World J Surg. 2016;40:510-5. 10.1007/s00268-015-3327-7 26546190

[28] 28. Ito Y, Tomoda C, Uruno T, Takamura Y, Miya A, Kobayashi K, et al. Preoperative ultrasonographic examination for lymph node metastasis: usefulness when designing lymph node dissection for papillary microcarcinoma of the thyroid. World J Surg. 2004;28:498-501. 10.1007/s00268-004-7192-z 15085396

[29] 29. Tomoda C, Sugino K, Matsuzu K, Uruno T, Ohkuwa K, Kitagawa W, et al. Cervical Lymph Node Metastases After Thyroidectomy for Papillary Thyroid Carcinoma Usually Remain Stable for Years. Thyroid; 2016;26:1706-11. 10.1089/thy.2016.0225 27616725

[30] 30. Robenshtok E, Fish S, Bach A, Domínguez JM, Shaha A, Tuttle RM. Suspicious cervical lymph nodes detected after thyroidectomy for papillary thyroid cancer usually remain stable over years in properly selected patients. J Clin Endocrinol Metab. 2012;97:2706-13. 10.1210/jc.2012-1553 22639292

[31] 31. Hay ID, Hutchinson ME, Gonzalez-Losada T, McIver B, Reinalda ME, Grant CS, et al. Papillary thyroid microcarcinoma: a study of 900 cases observed in a 60-year period. Surgery. 2008;144:980-987; discussion 987-8. 10.1016/j.surg.2008.08.035 19041007

[32] 32. Brito JP, Ito Y, Miyauchi A, Tuttle RM. A Clinical Framework to Facilitate Risk Stratification When Considering an Active Surveillance Alternative to Immediate Biopsy and Surgery in Papillary Microcarcinoma. Thyroid. 2016;26:144-9. 10.1089/thy.2015.0178 PMC4842944 26414743

[33] 33. Durante C, Montesano T, Torlontano M, Attard M, Monzani F, Tumino S, et al. Papillary thyroid cancer: time course of recurrences during postsurgery surveillance. J Clin Endocrinol Metab. 2013;98:636-42. 10.1210/jc.2012-3401 23293334

[34] 34. Giovanella L, Treglia G, Sadeghi R, Trimboli P, Ceriani L, Verburg FA. Unstimulated highly sensitive thyroglobulin in follow-up of differentiated thyroid cancer patients: a meta-analysis. J Clin Endocrinol Metab. 2014;99:440-7. 10.1210/jc.2013-3156 24285679

[35] 35. Malandrino P, Latina A, Marescalco S, Spadaro A, Regalbuto C, Fulco RA, et al. Risk-adapted management of differentiated thyroid cancer assessed by a sensitive measurement of basal serum thyroglobulin. J Clin Endocrinol Metab. 2011;96:1703-9. 10.1210/jc.2010-2695 21450986

